# Interactive Serious Game to Teach Basic Life Support Among Schoolchildren in Brazil: Design and Rationale

**DOI:** 10.2196/55333

**Published:** 2024-10-09

**Authors:** Uri Adrian Prync Flato, Emilio José Beffa dos Santos, Isabella Bispo Diaz T Martins, Vinicius Gazin Rossignoli, Thais Dias Midega, Lucas Kallas-Silva, Ricardo Ferreira Mendes de Oliveira, Adriana do Socorro Lima Figueiredo Flato, Mario Vicente Guimarães, Hélio Penna Guimarães

**Affiliations:** 1 Hospital Israelita Albert Einstein, Albert Einstein 626, 513, São Paulo, 05653120, Brazil, 55 11975858889; 2 Faculdade Israelita de Ciências em Saúde Albert Einstein, São Paulo, Brazil; 3 Internal Medicine Department, Universidade de Marília, Marília, Brazil; 4 Instituto Sanare, São Paulo, Brazil

**Keywords:** cardiopulmonary resuscitation, basic life support, serious game, CPR training, usability, cardiopulmonary, emergency, life support, CPR, training, education, game, gaming, educational, resuscitation, survey, satisfaction, SUS, user experience, System Usability Scale

## Introduction

Cardiovascular diseases are among the leading causes of death and morbidity worldwide [1,2]. Cardiopulmonary resuscitation (CPR) and early defibrillation increase survival chances [3]. Serious games (SGs) are tools used to enhance the learning process through entertainment. Current strategies focus on teaching CPR to the community and schoolchildren [4].

While other games exist for teaching basic life support (BLS), no studies have validated these for children in low- to middle-income settings. The SG Children Save Hearts teaches the 5 resuscitation steps per International Liaison Committee on Resuscitation (ILCOR) guidelines. Before use in schools, it requires a formal usability assessment by game developers and health care professionals to ensure ease of use, learning, and interaction.

The primary objective was to evaluate the usability of the SG Children Save Hearts among health care and IT professionals using the System Usability Scale (SUS) [5], a validated usability assessment tool.

## Methods

### Ethical Considerations

The study protocol was approved by the ethics committee of the University of Marília, Brazil (CAAE: 57160121400005496). All participants signed an informed consent form.

### Study Design

**Figure 1.**
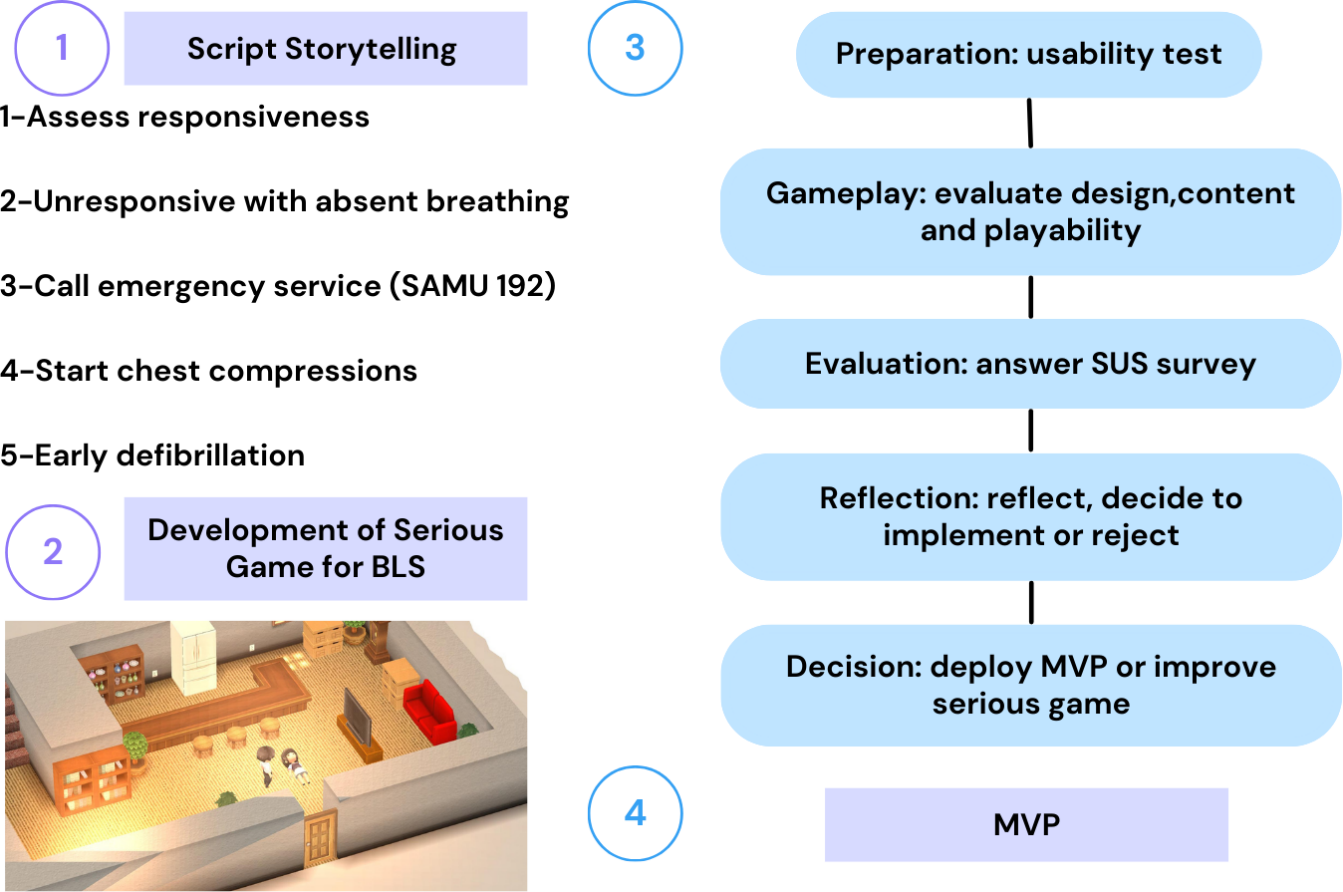
Design of a serious game for teaching cardiopulmonary resuscitation among schoolchildren. BLS: basic life support; MVP: minimum viable product; SAMU: Serviço de Atendimento Móvel de Urgência; SUS: System Usability Scale.

We used a nonprobabilistic casual sample to include IT and health care professionals. The usability test was conducted in August 2022 in the university’s IT department after a 10-minute lecture on the SG’s purpose. Children Save Hearts was developed on the Microsoft Smile Game Builder platform and implemented on Microsoft Windows (versions 7 to 11), targeting schoolchildren aged 7 to 17 years. The script and storytelling are based on the ILCOR 2020 guidelines. The game uses a joystick and simple commands to simplify the user experience (Multimedia Appendix 1). The design and testing process is illustrated in Figure 1.

After completing the SG, participants answered a 10-question survey on its usability using a Likert-type scale. The final grade was converted to a 0 to 100 scale (Multimedia Appendix 2). A grade above 70 was considered acceptable to proceed to a minimum viable product. A sample size of 17 users was required, based on a 10% estimated probability of encountering an interface error, to identify 85% of the problems.

### Statistical Analysis

Categorical variables are presented as absolute and relative frequencies. Continuous variables are presented as medians with IQRs. Normality was assessed with the Shapiro-Wilk test. Comparisons were made between IT and health care professionals. Continuous variables were compared using an independent 2-tailed *t* test (for normal distributions) or the Mann-Whitney *U* test (for nonnormal distributions). All analyses were performed using R (version 4.1.0; R Foundation for Statistical Computing).

## Results

Children Save Hearts was used by 17 volunteers with a median age of 22 (IQR 20‐26) years; 8 (47%) were male. Regarding professional training, 8 (47%) held a bachelor’s degree in IT and 9 (53%) were health care professionals. All participants played the game and answered the questionnaire. The median SUS score was 75 (IQR 72.5‐87.5; Table 1). Questions 2 and 4 had the lowest median scores, and questions 7 and 9 had the highest. Health care professionals gave higher grades to all 5 domains when compared to IT professionals. The average time spent in the game was 3.2 (SD 0.4) minutes.

**Table 1.** Participant characteristics and System Usability Scale (SUS) scores by profession.

	All (N=17)	IT professionals (n=8)	HCPs^a^ (n=9)	*P* value
**Participant characteristics**				
Age (years), median (IQR)	22.00 (20.00-26.00)	21.00 (18.75-22.75)	24.00 (22.00-27.00)	.07^b^
Male sex, n (%)	8 (47)	4 (50)	4 (44)	>.99^c^
**SUS scores, median (IQR)**				
Question 1	3.00 (3.00-4.00)	3.00 (2.00-3.25)	4.00 (3.00-4.00)	.08^b^
Question 2	2.00 (2.00-3.00)	2.00 (1.75-3.00)	3.00 (2.00-3.00)	.47^b^
Question 3	3.00 (3.00-4.00)	3.00 (3.00-4.00)	4.00 (3.00-4.00)	.36^b^
Question 4	2.00 (2.00-3.00)	2.00 (1.75-2.25)	2.00 (2.00-3.00)	.61^b^
Question 5	3.00 (3.00-4.00)	3.00 (3.00-3.00)	4.00 (3.00-4.00)	.07^b^
Question 6	4.00 (3.00-4.00)	3.00 (2.00-3.25)	4.00 (4.00-4.00)	.002^b^
Question 7	3.00 (3.00-4.00)	3.00 (3.00-3.00)	4.00 (4.00-4.00)	.009^b^
Question 8	4.00 (3.00-4.00)	3.00 (2.75-3.25)	4.00 (4.00-4.00)	.02^b^
Question 9	4.00 (3.00-4.00)	3.00 (3.00-3.25)	4.00 (4.00-4.00)	.03^b^
Question 10	3.00 (3.00-4.00)	3.50 (3.00-4.00)	3.00 (3.00-4.00)	.68^b^
**Analysis, median (IQR)**				
Total score	30.00 (29.00-35.00)	27.50 (25.50-30.50)	34.00 (30.00-35.00)	.05^d^
SUS grade	75.00 (72.50-87.50)	68.75 (63.75-76.25)	85.00 (75.00-87.50)	.05^d^
Score for ease of learning domain (Q3, Q4, Q7, Q10)	3.25 (2.75-3.25)	3.00 (2.50-3.25)	3.25 (2.75-3.50)	.54^d^
Score for efficiency domain (Q5, Q6, Q8)	3.67 (3.00-4.00)	3.00 (2.50-3.17)	4.00 (3.67-4.00)	.02^b^
Score for ease of memorization domain (Q2)	2.00 (2.00-3.00)	2.00 (1.75-3.00)	3.00 (2.00-3.00)	.47^b^
Score for minimization of errors domain (Q6)	4.00 (3.00-4.00)	3.00 (2.00-3.25)	4.00 (4.00-4.00)	.002^b^
Score for satisfaction domain (Q1, Q4, Q9)	2.67 (2.67-3.33)	2.67 (2.33-2.83)	3.33 (2.67-3.67)	.08^b^

a HCP: health care professional.

b Mann-Whitney *U* test.

c Fisher test.

d 2-sample Student *t* test.

## Discussion

We developed an SG, Children Save Hearts, to teach BLS to schoolchildren. When tested on 17 IT and health care professionals, it achieved an overall mean SUS score of 75, suitable for implementation.

Novel technologies like virtual reality (VR) have been successfully used in Europe to teach CPR to schoolchildren [6]. However, transferring this technology to limited-income countries faces challenges, such as language barriers, VR device acquisition, cultural context, and technical support. Previous SGs for teaching CPR were developed and tested in high-income countries [7]. Educational strategies for teaching CPR in limited-income countries have focused on health care professionals and students [8], not schoolchildren, highlighting a significant gap in the literature. This is the first SG developed in Brazil in Portuguese for schoolchildren.

Our study has some limitations. First, we had a small sample size due to insufficient data to calculate sample size in usability tests and financial constraints in contracting a software house. Continuous usability monitoring with larger sample sizes is needed to maintain external validation. Further studies should target schoolchildren to assess the effectiveness of teaching BLS in schools and explore user experiences to gain insights into how users feel about SGs.

Active teaching methods are crucial to improving survival rates and translating accessible knowledge into practice. Programs like Kids Save Lives [9] and World Restart a Heart Day [10] are teaching schoolchildren that CPR is vital. Despite some usability issues, the game is adequate for testing in schoolchildren.

## Supplementary material

10.2196/55333 Multimedia Appendix 1 Serious game screenshots and setup.

10.2196/55333 Multimedia Appendix 2 System Usability Scale (SUS) questions and domains.

## Abbreviations

BLS: basic life support
CPR: cardiopulmonary resuscitation
ILCOR: International Liaison Committee on Resuscitation
SG: serious game
SUS: System Usability Scale
VR: virtual reality
