# Supplementary figures and images for: Interactive Serious Game to Teach Basic Life Support Among Schoolchildren in Brazil: Design and Rationale
Source: JMIR Serious Games. 2024 Oct 9;12:e55333. doi: 10.2196/55333 (PMC11481818; doi:10.2196/55333)

## Slide 1
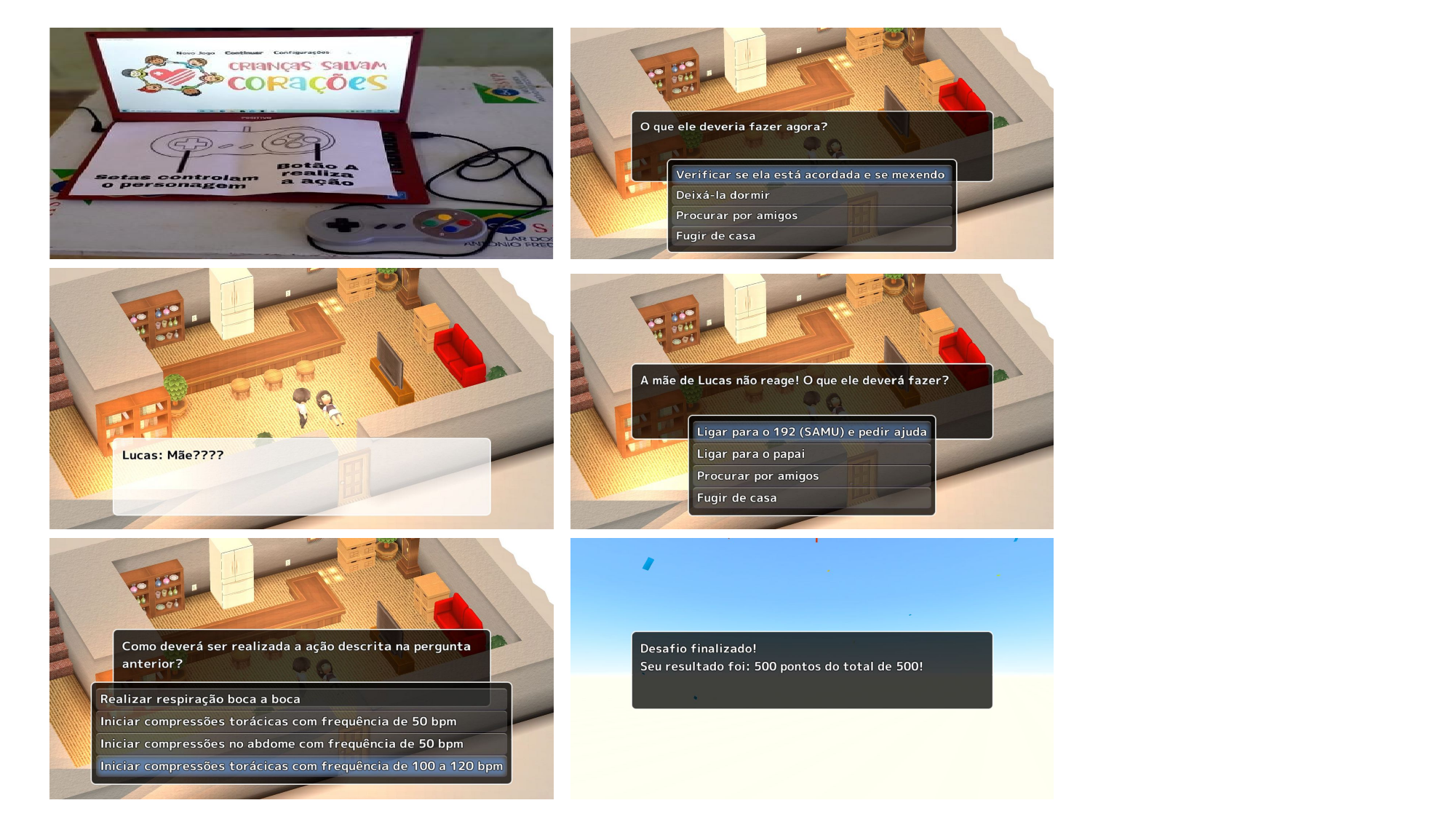

Supplement: Multimedia Appendix 1 [file games-v12-e55333-s001.pptx]
