# Supplementary material for: Interactive Serious Game to Teach Basic Life Support Among Schoolchildren in Brazil: Design and Rationale
Source: JMIR Serious Games. 2024 Oct 9;12:e55333. doi: 10.2196/55333 (PMC11481818; doi:10.2196/55333)
Supplement: Multimedia Appendix 2 [file games-v12-e55333-s002.docx]

**Multimedia Appendix 1: SUS Questionnaire questions and domains**

| **Item (a)** | **Questions** |
| --- | --- |
| Q1 | I think I'd like to use this system often |
| Q2 | I find the system unnecessarily complex |
| Q3 | I found the system easy to use |
| Q4 | I think I would need help from a person with technical knowledge to use the system |
| Q5 | I think the various functions of the system are very well integrated |
| Q6 | I think the system has a lot of inconsistency |
| Q7 | I imagine people will learn how to use this system quickly |
| Q8 | I found the system cluttered to use |
| Q9 | I felt confident when using the system |
| Q10 | I had to learn several new things before I could use the system |
| **Item** | **Domains (b)** |
| Q3, Q4, Q7, Q10 | Ease of learning |
| Q5, Q6, Q8 | Efficiency |
| Q2 | Ease of memorization |
| Q6 | Minimization of errors |
| Q1, Q4, Q9 | Satisfaction |

(a) Questions applied in the System Usability Scale (SUS). In each question, the user rates their experience by means of a *Likert* scale, assigning grades from 1 to 5, with grade 1 corresponding to "strongly disagree" and grade 5 corresponding to "strongly agree". After a conversion based on SUS, each question assumes a score from 0 to 4, with the number 4 corresponding to the best performance. The scores of each question are added up and, at the end, multiplied by 2.5, resulting in a scale ranging from 0 to 100. (b) A group of questions form a domain in the SUS. SUS consists of 5 domains.
